# Flow-Dependent Corrosion Behavior and Surface Degradation of X70 Pipeline Steel in Seawater Containing *Pseudomonas aeruginosa*

**DOI:** 10.3390/ma19061047

**Published:** 2026-03-10

**Authors:** Guiyuan Xie, Sixiang Lan, Yinghui Wang, Xingying Tang, Riguang Zhu, Ke Li, Pengwei Ren

**Affiliations:** 1 Guangxi Laboratory on the Study of Coral Reefs in the South China Sea, Coral Reef Research Center of China, School of Marine Sciences, Guangxi University, Nanning 530004, China; 2 Institute of Green and Low Carbon Technology, Guangxi Institute of Industrial Technology, Nanning 530200, China; 15901131316@163.com; 3 China Certification & Inspection Group Guangxi Co., Ltd., Nanning 530022, China

**Keywords:** *P. aeruginosa*, microbiologically influenced corrosion, flow-accelerated corrosion, X70 pipeline steel

## Abstract

The corrosion behavior of pipeline steels in marine environments is strongly affected by hydrodynamic conditions and microbial activity, yet their coupled influence remains insufficiently understood. In this study, the corrosion behavior of X70 pipeline steel was systematically investigated in flowing artificial seawater over a velocity range of 0–1.5 m/s, under both sterile conditions and in the presence of *Pseudomonas aeruginosa*. Corrosion weight loss measurements, electrochemical techniques, and surface characterization were employed to evaluate flow-dependent corrosion evolution. The results show that flow velocity plays a dominant role in regulating corrosion behavior. Under sterile conditions, increasing flow velocity enhances mass transfer and surface renewal, leading to progressively increased corrosion severity. In the presence of *P. aeruginosa*, corrosion behavior exhibits a non-monotonic dependence on flow velocity. Lower flow velocities are associated with reduced corrosion rates and relatively uniform surface degradation, whereas moderate flow velocities promote localized corrosion and increased pitting severity. At higher flow velocities, strong hydrodynamic effects suppress the retention of corrosion products and microbe-associated surface layers, resulting in corrosion behavior primarily controlled by fluid flow. Overall, the results indicate that microbial presence modifies the flow–corrosion relationship of X70 steel by altering interfacial conditions under low-to-moderate flow regimes.

## 1. Introduction

Pipeline steels are widely used as structural materials in oil and gas transportation systems due to their favorable mechanical properties and cost-effectiveness. Among them, X70 pipeline steel is commonly applied in marine and offshore environments, where long-term exposure to seawater can lead to corrosion-induced surface degradation and localized damage, thereby affecting the durability and performance of the material [1,2,3]. In chloride-containing marine environments, corrosion behavior is further influenced by multiple interacting factors, including hydrodynamic conditions and biological activity [4,5,6,7,8].

Seawater flow velocity is an important environmental parameter that directly affects the corrosion behavior of pipeline steels. Under flowing conditions, enhanced mass transfer of dissolved oxygen (DO) and aggressive ions can accelerate electrochemical reactions, while fluid-induced shear may remove corrosion products and expose fresh metal surfaces [9,10,11,12,13]. Previous studies have shown that increasing flow velocity generally promotes erosion–corrosion or flow-accelerated corrosion of carbon steels and pipeline steels [11,14,15,16]. However, the corrosion response does not increase monotonically with flow velocity, as the stability of corrosion product layers and surface degradation modes may vary depending on the balance between electrochemical reactions and mechanical scouring effects [9,17]. From a materials perspective, seawater flow not only alters the transport of corrosive species but also influences the evolution of surface morphology and localized corrosion on steel substrates [8,9,13].

In addition to hydrodynamic effects, microbial activity is recognized as an important factor affecting corrosion processes in marine environments. Microbiologically influenced corrosion (MIC) occurs when microorganisms colonize metal surfaces and modify the local interfacial environment through metabolic activity and biofilm formation [5,18]. Microbial biofilms can alter local chemistry near the metal surface, leading to changes in corrosion product formation and corrosion morphology [19,20,21,22]. While MIC has been extensively investigated under stagnant or low-flow conditions, increasing evidence suggests that microbial effects on corrosion behavior may persist under flowing seawater environments [23,24].

Most existing studies on MIC under flow conditions have focused on sulfate-reducing bacteria, whereas comparatively fewer investigations have addressed the corrosion behavior associated with facultative aerobic or nitrate-reducing bacteria [19,25]. *Pseudomonas aeruginosa*, a bacterium frequently detected in marine environments, has been reported to influence corrosion behavior through surface colonization and biofilm development [26,27,28]. However, current studies concerning *P. aeruginosa*-related corrosion are predominantly conducted under static or simplified laboratory conditions, and experimental data describing corrosion behavior and surface degradation under combined seawater flow and microbial presence remain limited [24,29,30].

Under flowing seawater conditions, seawater flow not only alters the transport of corrosive species but also influences the evolution of surface morphology and localized corrosion on steel substrates. [28]. Understanding how corrosion behavior varies across these flow conditions in both sterile and bacteria-containing seawater is essential for assessing corrosion risks and optimizing operational parameters. However, systematic experimental data comparing the corrosion behavior of X70 pipeline steel under different flow velocities, with and without microbial activity, are still limited.

In this study, the corrosion behavior of X70 pipeline steel was investigated under flowing artificial seawater over a velocity range of 0–1.5 m/s, both in the absence and presence of *P. aeruginosa*. Corrosion weight loss measurements, electrochemical tests, surface morphology observations, and corrosion product analyses were conducted to evaluate flow-dependent corrosion behavior and surface degradation characteristics. Unlike previous studies that primarily focused on either static MIC or single flow conditions, the present work systematically examines the transition of corrosion behavior across multiple flow regimes. Particular attention is given to identifying velocity-dependent shifts in dominant corrosion mechanisms and the competitive roles of biofilm stability and hydrodynamic shear. The findings aim to clarify how flow intensity regulates the balance between microbial activity and fluid-induced surface renewal under controlled low-oxygen laboratory conditions.

## 2. Materials and Methods

### 2.1. Materials, Microorganisms, and Test Solutions

Flow-induced corrosion experiments and subsequent testing and characterization were conducted using X70 pipeline steel coupons with dimensions of 10 mm × 10 mm × 3 mm. The main chemical composition of the X70 steel is listed in Table 1.

Prior to the flow-induced corrosion experiments, the working surfaces of the coupons were sequentially ground and polished using 400, 800, 1200, and 2000 grit SiC sandpapers, followed by a 1 μm diamond suspension. The specimens were then cleaned with sterile distilled water, acetone, and anhydrous ethanol, and finally sterilized by exposure to ultraviolet light for 30 min. For electrochemical measurements, a copper wire was welded to the back of each coupon to serve as the electrical connection. The remaining surfaces were sealed with epoxy resin, leaving a 1 cm^2^ working area exposed for corrosion testing.

The *P. aeruginosa* strain used in this study was provided by the South China Sea Institute of Oceanology, Chinese Academy of Sciences, and was originally isolated from coastal seawater of the South China Sea (strain 1A05429). The bacteria were stored at −80 °C in liquid nitrogen after being encapsulated in glycerol. Prior to use, the strain was revived in LB broth. The main components of the LB liquid medium are as follows: 0.5 g/L K_2_HPO_4_, 0.5 g/L NaNO_3_, 0.2 g/L CaCl_2_, 0.5 g/L MgSO_4_·7H_2_O, 0.5 g/L (NH_4_)_2_SO_4_, and 10.0 g/L ferric ammonium citrate. Artificial seawater was prepared to simulate the marine environment according to ASTM D1141-98 [31]. The composition of the artificial seawater included: 24.48 g/L NaCl, 3.916 g/L Na_2_SO_4_ 1.16 g/L CaCl_2_, 0.695 g/L KCl, 5.2 g/L MgCl_2_, 0.201 g/L NaHCO_3_, 0.027 g/L H_3_BO_3_, 0.025 g/L SrCl_2_, 0.101 g/L KBr, and 0.003 g/L NaF. The pH of the solution was adjusted to 7.2–7.5 using 1 mol/L NaOH, followed by autoclaving at 121 °C for 30 min to eliminate microbial contamination.

After 24 h of bacterial cultivation, 3000 mL of sterilized artificial seawater containing 10% *P. aeruginosa* LB broth was added to the sterile reactor. According to our previous plate counting results, the bacterial density in similar systems typically stabilizes at 10^6^ to 10^7^ CFU/mL in the exposure medium after inoculation. In the sterile control group, 3000 mL of artificial seawater containing 10% sterile LB broth was used. In the inoculated group, 3000 mL of artificial seawater with 10% LB broth containing *P. aeruginosa* was used, and the dissolved oxygen concentration in the solution was regulated using a gas cylinder. All corrosion experiments were carried out under sealed conditions. The reactors were immersed in a thermostatic water bath to maintain a constant temperature of 25 °C. Flow-induced corrosion tests were performed for 7 days, with each coupon tested in triplicate. All experiments in this study followed the same inoculation and cultivation protocol to ensure consistency under different flow rate conditions. The presented data are representative, and the observed trends were fully reproducible across repeated experiments.

### 2.2. Experimental Setup

**Figure 1:**
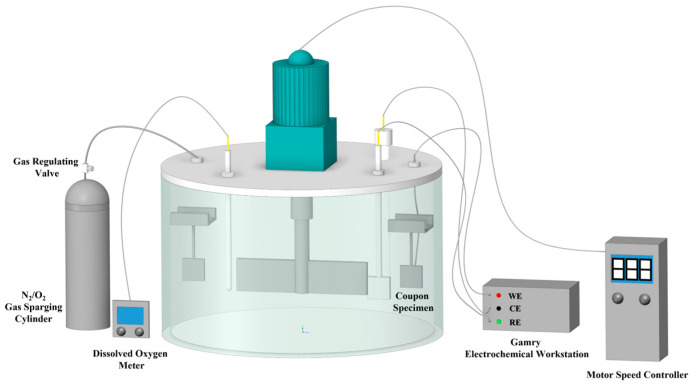
Schematic diagram of the experimental setup for seawater scouring.

Based on an existing deep-sea flow-induced corrosion setup, a miniature seawater flow reactor was designed and fabricated in-house to perform laboratory simulations. A schematic of the experimental apparatus is shown in Figure 1 [32]. The motor speed of the reactor was controlled in the range of 0–400 r/min, corresponding to a flow velocity of 0–2.0 m/s. The linear velocity at the cam surface was calculated based on the rotational speed and the radial distance from the shaft center, and the selected velocity range (0–1.5 m/s) was achieved by adjusting the motor speed accordingly. Four coupon suspension points were installed inside the reactor, and the system was directly connected to an external electrochemical workstation, enabling controlled electrochemical measurements to be performed in situ in the test medium, thereby improving the accuracy and reliability of the corrosion data. In addition, the reactor was connected to both an external oxygen cylinder and a dissolved oxygen meter. The DO concentration in the medium under different flow conditions was regulated and monitored by adjusting the oxygen supply rate. Under sterile laboratory conditions, the measured DO concentration of the seawater used for corrosion tests was 2.0 ± 0.2 ppm, ensuring controlled and reproducible oxygen conditions during the experiments.

### 2.3. Electrochemical Test

Electrochemical measurements were performed using a Gamry electrochemical workstation, including open-circuit potential (OCP), electrochemical impedance spectroscopy (EIS), linear polarization resistance (LPR), and potentiodynamic polarization (PDP) tests under both bacterial and sterile conditions at different flow velocities. All electrochemical measurements were conducted using a CHI660D electrochemical workstation in a three-electrode configuration. In this system, a sealed X70 pipeline steel coupon served as the working electrode (WE), a platinum electrode as the counter electrode (CE), and an Ag/AgCl electrode as the reference electrode (RE). EIS measurements were performed at the OCP, with a frequency range of 10^5^–10^−2^ Hz. The OCP fluctuation over 500 s did not exceed 5 mV, and an AC perturbation amplitude of 5 mV was applied. The EIS data were analyzed and fitted using ZSimpWin software (Princeton Applied Research, Oak Ridge, TN, USA, https://www.ameteksi.com/products/software/zsimpwin, accessed on 4 February 2026). Potentiodynamic polarization tests were conducted over a potential range of −0.5 V to +0.5 V versus OCP, with the scan proceeding from cathodic to anodic potentials at a rate of 1 mV/s. A narrow scanning window of ±10 mV relative to the OCP was selected to closely approximate the true electrochemical state of the system. Polarization resistance (*R*_p_) was determined from the slope of the potential versus current curve, while the corrosion current density (*I*_corr_) was obtained from the PDP curves.

### 2.4. Surface Morphology Analysis

The surface morphology of the coupons was examined using an optical microscope (Nikon Eclipse LV150NL, Tokyo, Japan) and a scanning electron microscope (SEM, Hitachi SU5000, Chiyoda City, Japan). Prior to observation, corrosion coupons from bacterial experiments were fixed in 2.5% (*v*/*v*) glutaraldehyde at 4 °C for 8 h to preserve the biofilm. The samples were then rinsed twice with phosphate-buffered saline (PBS) and deionized water, followed by sequential dehydration in 25%, 50%, 75%, and 100% (*v*/*v*) ethanol for 10 min each. Finally, the samples were dried under a nitrogen stream and stored in a desiccator [33]. Corrosion coupons under sterile conditions were prepared using the same rinsing and drying procedures. Energy-dispersive X-ray spectroscopy (EDS, FEI TeN-Cai G2 f20 s-twin, Hillsboro, OR, USA) was employed at an accelerating voltage of 200 kV to analyze the elemental composition, distribution, and relative abundance of the corrosion products. After surface morphology and product analysis, the adherent biofilm and corrosion products were removed from the coupon surfaces according to ASTM G1-03 [34]. Optical microscopy was then used to identify the locations of maximum pit depth, which were subsequently used to determine the most severe localized corrosion. The pit morphology and maximum pit depth were further examined and measured using a confocal laser scanning microscope (CLSM, Model C2 Plus, Nikon, Tokyo, Japan).

### 2.5. Weight Loss Analysis

Before the corrosion tests, the length, width, and thickness of each coupon were precisely measured using a vernier caliper to calculate the effective surface area. The coupons were then rinsed with distilled water and absolute ethanol, blown dry with nitrogen gas, and weighed using a high-precision electronic analytical balance (model MCE125P-2CCN-U, accuracy 0.01 mg). Each coupon was weighed at least four times to obtain an accurate initial mass. After completion of the corrosion tests under the respective conditions, the coupons were removed, rinsed thoroughly with deionized water, and then immersed in a rust remover solution prepared according to GB/T 16545-2015 standard [35]. (50 mL 36% HCl + 50 mL deionized water + 0.346 g hexamethylenetetramine) for 30–60 s under ultrasonic cleaning to remove corrosion products. Subsequently, the coupons were rinsed again with distilled water and absolute ethanol, dried with nitrogen gas, and then weighed once more after complete drying. Corrosion rates were calculated using Equation (1). For each corrosion condition, three parallel coupons were tested and the average corrosion rate was reported.

(1) $$\nu =(m_{0}-m_{1})/\mathrm{St}$$

where ν is the corrosion rate (g·m^−2^·h^−1^), m_0_ and m_1_ are the initial and final weights of the specimen (g), respectively, t is the immersion time (h), and S is the exposed surface area (m^2^).

## 3. Results

Surface morphology under static immersion (0 m/s) has been comprehensively re-ported in our previous study (https://doi.org/10.1016/j.matchemphys.2025.130478, accessed on 2 March 2026). To avoid duplication of previously published material, static SEM/EDS images and electro-chemical measurements are not reproduced here. The present work focuses on the evolution of corrosion behavior under flow conditions.

### 3.1. Subsection

**Figure 2:**
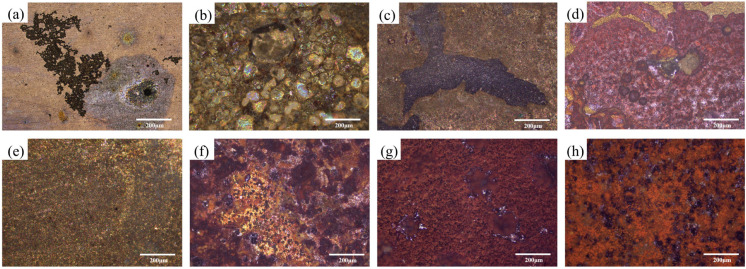
Metallographic corrosion morphologies of X70 steel under different conditions after 7 days of scouring. Sterile seawater: (**a**): 0.3 m/s, (**b**): 0.5 m/s, (**c**): 1.0 m/s, (**d**): 1.5 m/s. *P. aeruginosa* seawater: (**e**): 0.3 m/s, (**f**): 0.5 m/s, (**g**): 1.0 m/s, (**h**): 1.5 m/s.

Figure 2 shows the metallographic corrosion morphologies of X70 steel after 7 days of flow-induced corrosion under different flow velocities. Figure 2a–d show the morphologies in sterile seawater at various flow velocities. At a flow velocity of 0.3 m/s, the steel surface exhibited almost no distinct corrosion products; only an irregularly distributed corrosion product film could be observed, accompanied by the formation of large and deep pits. With increasing flow velocity, the surface corrosion products of X70 steel became more complex, and both the size and depth of erosion pits increased. The corrosion product film displayed a stratified structure, with its color gradually shifting from yellow-brown to reddish-brown, while the overall corrosion mode transitioned toward general corrosion. Figure 2e–h illustrate the morphologies in seawater inoculated with *P. aeruginosa* after 7 days of scouring at different flow velocities. At 0.3 m/s, a dense corrosion product film formed on the steel surface, with only fine pits being observed. As the flow velocity increased, the amount of corrosion products increased, and these deposits appeared porous and loosely adherent, with a characteristic reddish-brown and black coloration. Comparison of results under the same flow velocity between sterile and bacterial conditions indicates that the presence of *P. aeruginosa* not only accelerated the transformation of corrosion products on the X70 steel surface but also promoted a more uniform distribution of these products.

**Figure 3:**
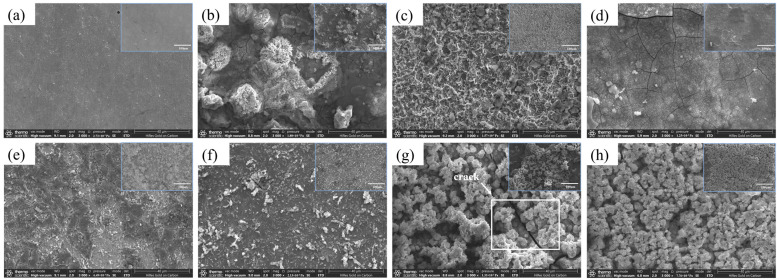
SEM morphologies of X70 steel under different conditions after 7 days of scouring. Sterile seawater: (**a**): 0.3 m/s, (**b**): 0.5 m/s, (**c**): 1.0 m/s, (**d**): 1.5 m/s. *P. aeruginosa* seawater: (**e**): 0.3 m/s, (**f**): 0.5 m/s, (**g**): 1.0 m/s, (**h**): 1.5 m/s.

Figure 3 shows the SEM morphologies of X70 steel after 7 days of flow-induced corrosion under different conditions. Figure 3a–d depict the surface microstructures of X70 steel in sterile seawater at varying flow velocities. At 0.3 m/s, the steel exhibited almost no corrosion, with surface scratches still visible and only a few localized pits present. At flow velocities of 0.5–1.0 m/s, the steel surface was completely covered by corrosion products, which formed nest-like regions composed predominantly of needle- or rod-shaped and flake-like structures [32], morphologically consistent with γ-FeOOH and β-FeOOH reported in the previous literature [36,37]. At 1.5 m/s, the corrosion product layer was more uniformly distributed, displaying numerous cotton-ball-like structures with obvious surface cracks, characteristic of α-FeOOH [37]. Figure 3e–h show the SEM morphologies of X70 steel after 7 days of flow-induced corrosion in seawater containing *P. aeruginosa* at different flow velocities. The surface morphologies under bacterial conditions exhibited distinct features compared with sterile conditions. Under the influence of microbiologically influenced corrosion, biofilms composed of surface bacteria and extracellular polymeric substances overlapped with metallic compounds to form composite product layers, which were clustered on the steel surface [29]. At 0.3 m/s, numerous cracks were observed on the steel surface, with only a small amount of bacterial cells and biofilm coverage, and corrosion products were scarce. When the flow velocity exceeded 0.3 m/s, the mass transfer of dissolved oxygen and nutrients in water was enhanced, promoting the aggregation of *P. aeruginosa* on the steel surface. As shown in Figure 3f, abundant rod-shaped *P. aeruginosa* and EPS were observed on the corrosion product layer [19,20]. The steel surface became covered with a composite layer formed by the accumulation of metallic compounds and biofilm. The metabolic activities of *P. aeruginosa* rendered the corrosion product layer loose and porous, facilitating the formation of cracks and voids. The initiation and propagation of these cracks may be attributed to internal physical growth stresses arising from volumetric changes associated with phase transformations of metabolic products during bacterial growth [21,22].

### 3.2. Elemental Composition and Structure Analysis

**Figure 4:**
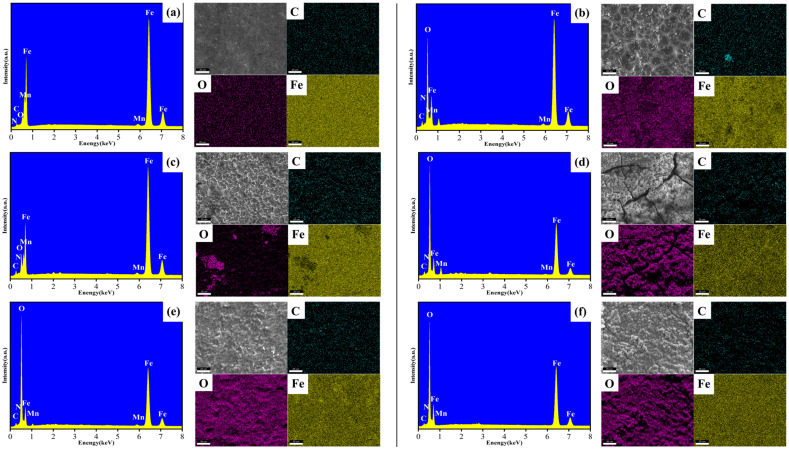
Surface element content of X70 steel under different conditions after 7 days of scouring. Sterile seawater: (**a**): 0.3 m/s, (**b**): 1.0 m/s, (**c**): 1.5 m/s; *P. aeruginosa* seawater: (**d**): 0.3 m/s, (**e**): 1.0 m/s, (**f**): 1.5 m/s.

**Figure 5:**
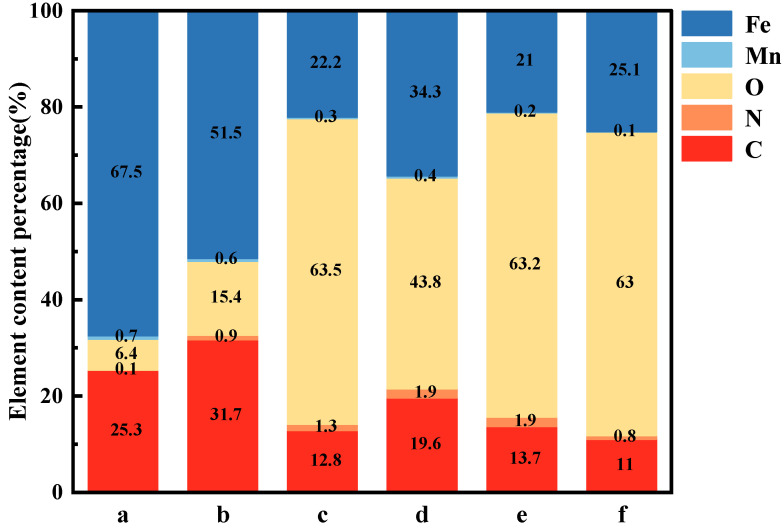
Comparison of surface element content variations in X70 steel under different conditions after 7 days of scouring. Sterile seawater: (**a**): 0.3 m/s, (**b**): 1.0 m/s, (**c**): 1.5 m/s. *P. aeruginosa* seawater: (**d**): 0.3 m/s, (**e**): 1.0 m/s, (**f**): 1.5 m/s.

Figure 4 presents the EDS maps of X70 steel after 7 days of flow-induced corrosion under different conditions. Based on the elemental distributions of the corrosion products, the comparative changes in surface elemental contents are summarized in Figure 5. Under all seawater conditions, Fe and O were the most abundant elements, indicating that the corrosion products primarily consisted of iron oxides or hydroxides. In sterile seawater, the percentages of Fe and O remained high. With increasing flow velocity, the Fe content decreased while the O content increased, suggesting that flow-induced scouring accelerated the corrosion of X70 steel, resulting in a surface rich in iron oxides or hydroxides. Notably, at lower flow velocities, the C content on the steel surface was relatively high. Image overlay analysis revealed that C was distributed randomly across the surface: at 0.3 m/s, C was mainly located within surface scratches, whereas at 1.0 m/s, C accumulated on the ridges surrounding pits. The relatively high Fe content at this stage indicates that some corrosion products had formed but were removed by scouring, exposing the underlying steel substrate. In seawater containing *P. aeruginosa*, the C content was lower than that in the sterile seawater environment. With increasing flow velocity, the C content gradually decreased. Meanwhile, under bacterial conditions, the O content remained at a relatively high level, whereas the Fe content was comparatively lower, suggesting that *P. aeruginosa* may utilize Fe and promote oxidation on the surface of X70 steel. A significant difference in surface carbon content was observed between the sterile and bacterial environments. However, considering the limitations of energy-dispersive spectroscopy in the quantitative analysis of light elements, as well as the possible influence of organic residues or biofilm components, the observed variation in carbon content cannot be conclusively attributed to a specific factor.

### 3.3. Pit Morphology and Depth Analysis

**Figure 6:**
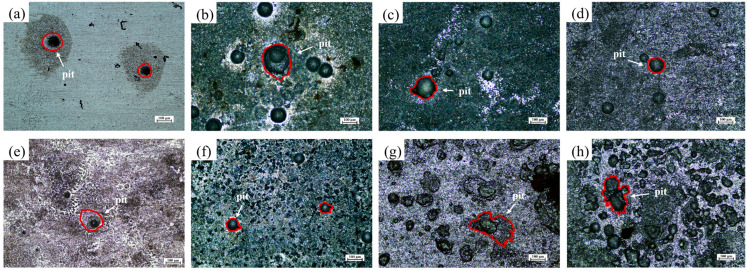
Corrosion pit morphology of X70 steel after removal of corrosion products after 7 days of scouring under different conditions; Sterile seawater: (**a**): 0.3 m/s, (**b**): 0.5 m/s, (**c**): 1.0 m/s, (**d**): 1.5 m/s. *P. aeruginosa* seawater: (**e**): 0.3 m/s, (**f**): 0.5 m/s, (**g**): 1.0 m/s, (**h**): 1.5 m/s.

**Figure 7:**
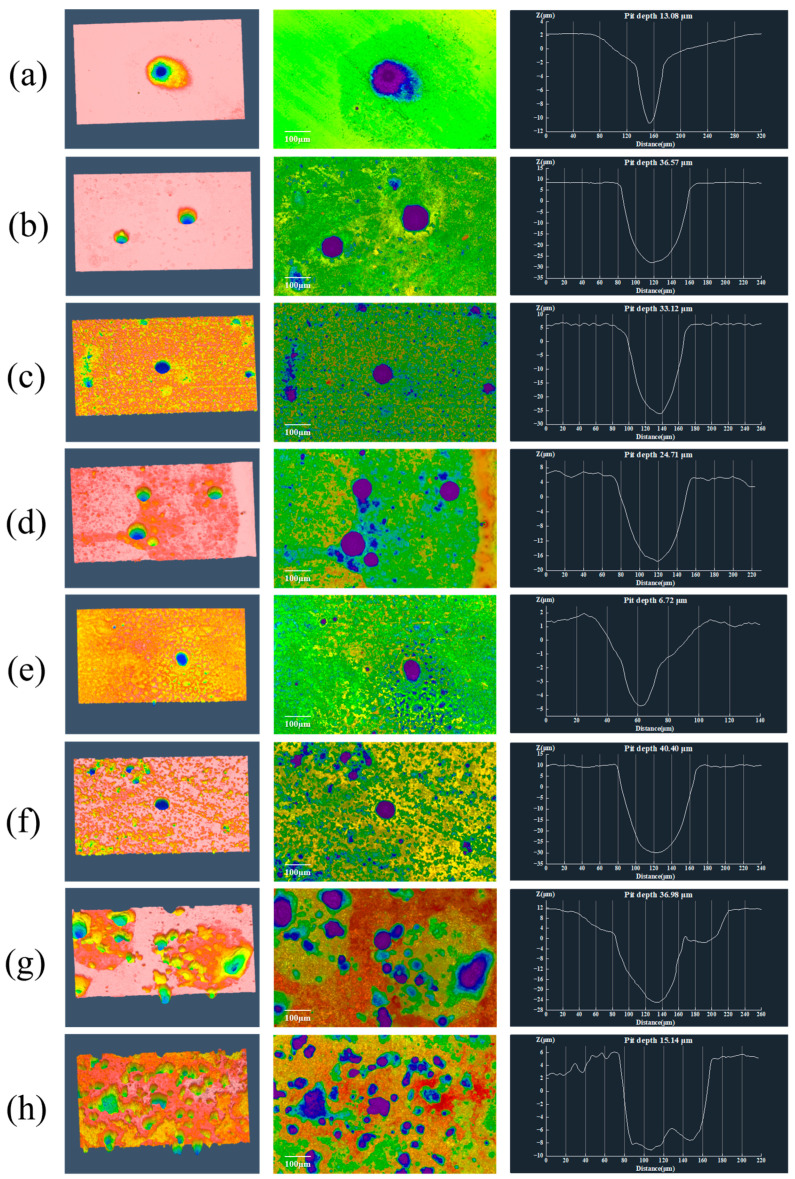
Three-dimensional topography and roughness curve of corrosion pits on X70 steel surface after 7 days of scouring under different conditions. Sterile seawater: (**a**): 0.3 m/s, (**b**): 0.5 m/s, (**c**): 1.0 m/s, (**d**): 1.5 m/s. *P. aeruginosa* seawater: (**e**): 0.3 m/s, (**f**): 0.5 m/s, (**g**): 1.0 m/s, (**h**): 1.5 m/s. (The color scale represents the relative surface height, where warmer colors indicate higher regions and cooler colors indicate lower regions).

Figure 6 presents the metallographic images of the maximum pitting sites on X70 steel after removal of surface corrosion products under different seawater flow conditions. The surface pitting morphologies were further analyzed using a laser confocal scanning microscope, and Figure 7 displays the three-dimensional morphology and surface roughness profiles of the most severe pits under various experimental conditions. As observed from the figures, after exposure to sterile seawater, the size and number of maximum pits on the X70 steel surface first increased and then decreased with increasing flow velocity. In addition to the presence of relatively large pits, the 3D surface morphology shows that the overall surface remained relatively smooth. The maximum pit depth increased from 13.08 μm at 0.3 m/s to 36.57 μm at 0.5 m/s, then decreased to 24.71 μm at 1.5 m/s. Under static conditions (0 m/s), the maximum pit depth was 20.66 μm. These results suggest that moderate flow can exacerbate localized and uniform corrosion on X70 steel. The influence of flow velocity on corrosion type is multifaceted. On the one hand, flowing seawater facilitates the transport of corrosive species such as Cl^−^ to the metal surface, accelerating anodic dissolution [38] and promoting pitting corrosion. Additionally, flow alters the local stress environment around the pits, deepening the severity of pitting. On the other hand, higher flow velocities tend to dislodge corrosion product films from the steel surface [17], exposing the underlying substrate and thereby accelerating further corrosion.

In the seawater environment containing *P. aeruginosa*, the variation in the maximum pitting depth of X70 steel with seawater flow velocity is similar to that in sterile seawater. However, at flow velocities of 0.3 m/s and 1.5 m/s, the maximum pitting depth of X70 steel in sterile seawater is greater than that in the presence of *P. aeruginosa*, with the deepest pit reaching 40.40 μm at 0.5 m/s. According to the three-dimensional morphology, at higher flow velocities, the surface of X70 steel becomes rough and porous, and the entire observation area exhibits complex pits. These irregular pits are mainly caused by the aggregation of *P. aeruginosa* and the biofilm formation, which subsequently induces accelerated corrosion. At a flow velocity of 1.5 m/s, the maximum pit depth decreased to 15.14 μm, which was significantly shallower than that observed under sterile conditions at the same flow rate. This is attributed to the high flow velocity hindering the accumulation of *P. aeruginosa* on the X70 steel surface, whereby the microorganisms primarily acted to inhibit scouring-induced corrosion.

### 3.4. Electrochemical Results

**Figure 8:**
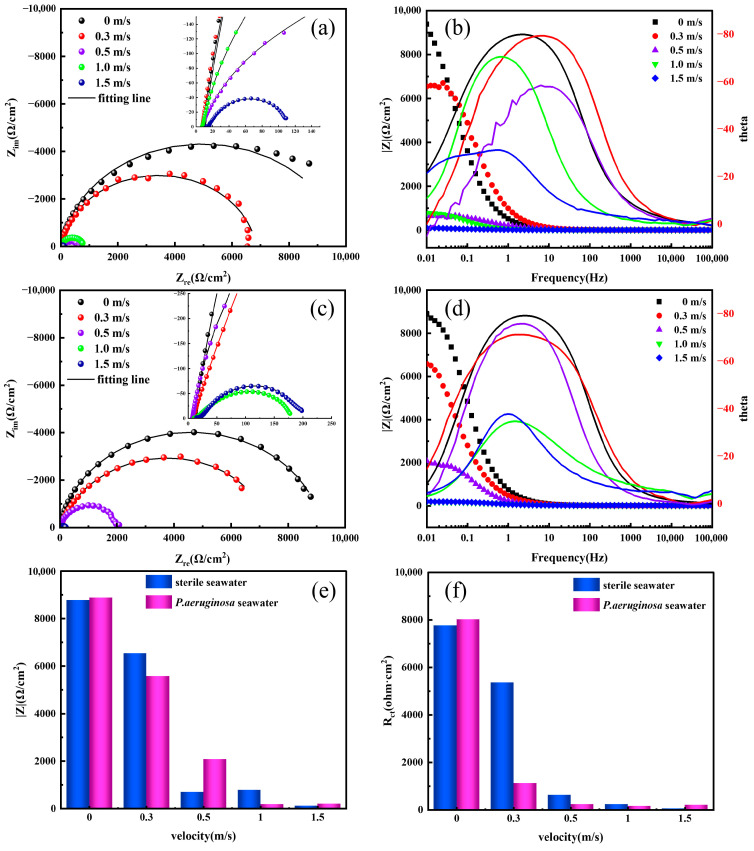
Impedance spectra and Bode-phase angle plots of X70 steel after 7 days of scouring under different conditions. (**a**): impedance spectra of sterile seawater, (**b**): Bode-phase angle of sterile seawater, (**c**): impedance spectra of *P. aeruginosa* seawater, (**d**): Bode-phase angle of *P. aeruginosa* seawater, (**e**): impedance modulus in the low-frequency region (|Z|0.01 Hz), (**f**): charge-transfer resistance, *R*_ct_.

**Figure 9:**
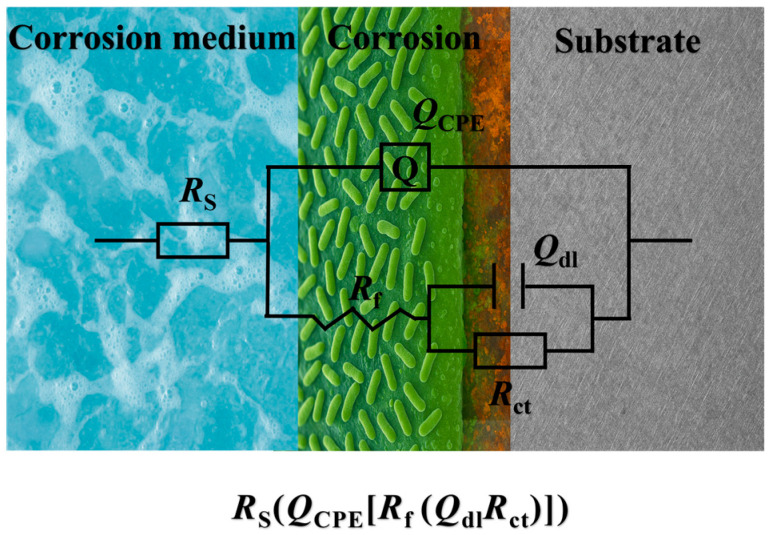
Equivalent circuits used for fitting the impedance spectra of X70 steel.

Figure 8 shows the impedance spectra and Bode phase angle plots of X70 steel after 7 days of seawater flow corrosion at different velocities, with and without *P. aeruginosa*. In sterile seawater flow conditions, the capacitive arc of X70 steel gradually decreased as the flow velocity increased, indicating a reduction in the overall resistance of the electrochemical system and a weakening of the material’s corrosion resistance. This suggests that flow erosion accelerates the corrosion process, leading to an increased corrosion rate. In seawater containing *P. aeruginosa*, the radius of the impedance spectrum decreased with increasing flow velocity up to 1.0 m/s, but slightly increased at 1.5 m/s. Correspondingly, the Bode plots show that the impedance modulus at low frequency (|Z| 0.01 Hz) follows a similar trend to the capacitive arc. A larger capacitive arc diameter and higher low-frequency impedance modulus generally indicate better corrosion resistance [39]. As shown in Figure 8b,d,e, the low-frequency impedance modulus in both flow conditions decreased with increasing flow velocity and dropped sharply at 0.5 m/s. When the flow velocity increased from 0 m/s to 1.5 m/s, the impedance modulus decreased from approximately 8800 Ω·cm^2^ to 115 Ω·cm^2^. All flow-exposed samples exhibited two time constants, attributed to the formation of surface films and charge transfer processes [40]. Of these, the high-frequency time constant corresponds to the capacitive and resistive behavior within the surface film, while the low-frequency time constant reflects the charge transfer processes at the metal/electrolyte or metal/film interface. Therefore, the equivalent circuit widely used for passivation systems (Figure 9) was selected to fit the impedance data. The resistance values were calculated according to the fitting formulas reported in previous work [41]. The equivalent circuit model consists of electrolyte resistance (*R*_s_), oxide film resistance or a combined resistance of the oxide film and biofilm (*R*_f_), charge transfer resistance (*R*_ct_), constant phase element representing the capacitance of the oxide layer (*Q*_CPE_), and double layer capacitance at the film/solution interface (*C*_dl_). The fitting results are summarized in Table 2, where the fitted curves closely match the experimental data with fitting errors less than 10%. Constant phase elements (CPE, denoted as *Q*) were used instead of ideal capacitors to account for surface heterogeneity and non-ideal interfacial behavior.

The charge transfer resistance (*R*_ct_) is inversely proportional to the corrosion rate. As shown in Table 2, *R*_ct_ decreases with increasing flow velocity in both sterile and bacteria-containing seawater environments. Before 0.3 m/s, the *R*_ct_ of X70 steel in the seawater environment containing *P. aeruginosa* is higher than that in sterile seawater, indicating that the corrosion resistance of X70 steel in the presence of *P. aeruginosa* is stronger than that in sterile seawater. The reason is that corrosive ions in seawater accelerate the corrosion of X70 steel, whereas in the seawater environment with *P. aeruginosa*, the biofilm formed by the bacteria can resist both the scouring effect of seawater flow and the attack of corrosive ions, thereby effectively mitigating the erosion of seawater on X70 steel. This explanation can be further verified by the electrode surface roughness parameter n shown in Table 2. When the flow velocity increases from 0.3 m/s to 1.0 m/s, *R*_ct_ decreases significantly, indicating a reduction in corrosion resistance. Moreover, *R*_ct_ in the bacterial seawater is lower than that in sterile seawater under these conditions, suggesting that *P. aeruginosa,* together with seawater flow, synergistically accelerates the corrosion of X70 steel. However, at the highest tested velocity of 1.5 m/s, *R*_ct_ in the bacterial seawater becomes higher than that in sterile seawater, implying that the biofilm again improves corrosion resistance at this flow rate. For both corrosive environments, the electrode surface roughness (n) in bacterial seawater is greater than that in sterile seawater, indicating a smoother electrode surface in the presence of *P. aeruginosa*. This is likely due to the conductive biofilm covering the steel surface, which effectively smooths the electrode interface [42].

**Figure 10:**
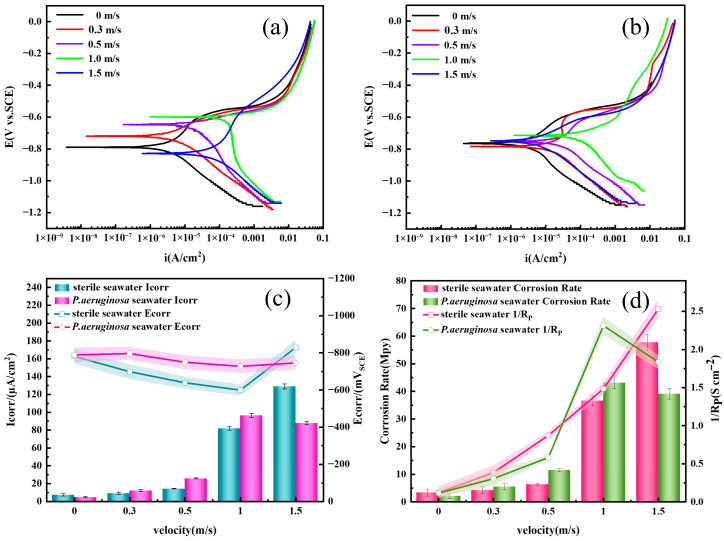
Potentiodynamic polarization curves of X70 steel under different conditions after 7 days of scouring and the corresponding fitted parameters. (**a**): Sterile seawater, (**b**): *P. aeruginosa* seawater, (**c**): Corrosion current (*I*_corr_) and Corrosion potential (*E*_corr_), (**d**): Corrosion rate and 1/*R*_P_.

Figure 10 shows the potentiodynamic polarization curves of X70 steel after 7 days of flow-induced corrosion at different flow velocities. The polarization resistance (*R*_p_) was calculated using the previously reported formula [41], and the results are summarized in Table 3. As seen in the figure, both the cathodic and anodic polarization curves contract toward the center with increasing flow velocity, indicating pronounced electrode depolarization. Correspondingly, corrosion current density increases while polarization resistance *R*_p_ (*R*_p_ = *R*_s_ + *R*_f_ + *R*_ct_) decreases, demonstrating that seawater flow accelerates the corrosion of X70 steel. This result is consistent with the trends observed in corrosion weight loss measurements.

The polarization curves of X70 steel in sterile seawater exhibit clear active corrosion behavior. Within the flow velocity range of 0–1.0 m/s, as the flow velocity increases, the shear force becomes stronger, and the corrosion product film is stripped away by the flowing seawater, causing the polarization curves to shift to the right. This is manifested as a positive shift in corrosion potential and an increase in current density. The reason is that the increase in flow velocity enhances the diffusion and transport of dissolved oxygen in seawater, thereby weakening electrode polarization and promoting the electrochemical corrosion of X70 steel, indicating that X70 steel is in an active dissolution state [43]. During this process, the corrosion current density increases from 7.49 µA/cm^2^ to 128.96 µA/cm^2^ and the electrochemical corrosion rate rises from 3.34 mpy to 57.62 mpy. When the flow velocity reaches 1.5 m/s, an apparent anodic plateau is observed in the polarization curve. However, considering the negative shift in *E*_corr_ and the relatively high corrosion current density, this feature does not represent true electrochemical passivation but rather a transient stabilization of the surface film under high flow disturbance. [44].

Under the condition of seawater flow with *P. aeruginosa*, a distinct transition region appears in the anodic branch at a flow velocity of 0.3 m/s, which may be attributed to surface state activation induced by *P. aeruginosa*. At flow velocities of 0.5–1.5 m/s, the polarization curves of X70 steel in seawater containing *P. aeruginosa* exhibit similar shapes, indicating that the corrosion mechanism remains essentially consistent [45]. This suggests that under these conditions, the flow velocity does not alter the electrode reaction, and the electrochemical corrosion of X70 steel is mainly regulated by *P. aeruginosa*. The corrosion potential (*E*_corr_) shows little variation, whereas the corrosion current density (*I*_corr_) generally increases with increasing flow velocity. When the flow velocity is below 0.3 m/s or above 1.5 m/s, the corrosion current density in bacteria-containing seawater is lower than that in sterile seawater; the opposite trend occurs at flow velocities between 0.5 and 1.0 m/s. This behavior is attributed to the biofilm formed by *P. aeruginosa* on the steel surface, which partially shields X70 steel from erosion under low flow velocities, effectively mitigating flow-induced corrosion. However, higher flow velocities enhance the diffusion and transport of oxygen and nutrients in seawater, promoting microbial growth and proliferation. Moreover, flow-induced erosion roughens the steel surface, providing favorable attachment sites for *P. aeruginosa*. Consequently, free-floating bacteria can accumulate near erosion pits, accelerating localized pitting corrosion through the coupling of flow erosion and microbiologically influenced corrosion. At a flow velocity of 1.5 m/s, the hydrodynamic force of seawater exceeds the adhesion strength of the biofilm, preventing its stable formation on the steel surface. Notably, polarization resistance (*R*_p_) increases at this velocity, indicating that the biofilm still offers some protection to the substrate metal by impeding seawater erosion and weakening microbial corrosion effects [46].

### 3.5. Weight Loss Results

**Figure 11:**
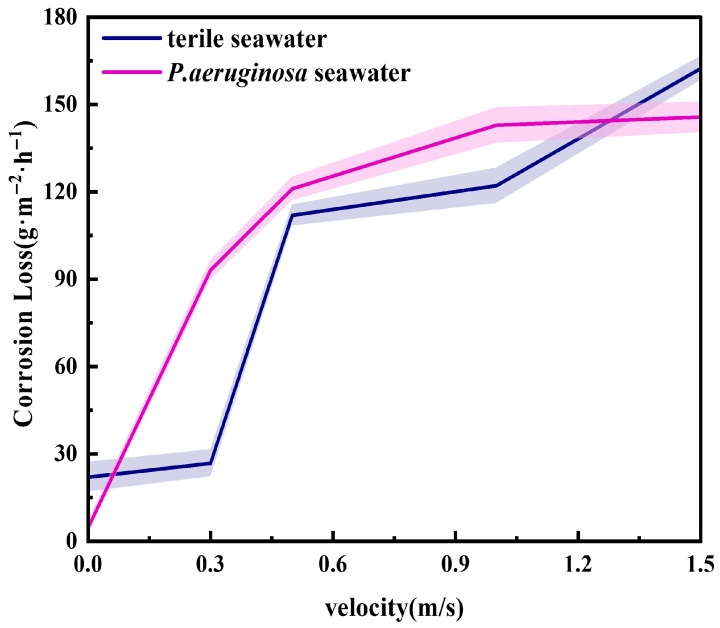
Corrosion weight loss rate of X70 steel after 7 days of scouring under different conditions. (The shaded band denotes the standard deviation from three independent measurements, n = 3 samples).

The corrosion weight loss of X70 steel after exposure to seawater with and without *P. aeruginosa* under different flow velocities is shown in Figure 11. Comparing the corrosion weight loss rates under static immersion conditions, it is evident that the corrosion weight loss rates of X70 steel increase with increasing flow velocity in both sterile and bacteria-containing seawater. This indicates that seawater flow accelerates the corrosion of X70 steel.

In the sterile seawater flow environment, the corrosion weight loss rate increased from 21.96 g·m^−2^·h^−1^ to 162.32 g·m^−2^·h^−1^ as the flow velocity increased, representing nearly an eightfold increase. The corrosion weight loss rate exhibits a significant change at 0.5 m/s, which is attributed to the accelerated mass transfer of the corrosive medium with increasing flow velocity, thereby enhancing the erosion effect on the X70 steel surface [14]. At higher flow velocities, mechanical wear leads to the rupture of the oxide film, aggravating the erosion–corrosion process [15,16]. In seawater containing *P. aeruginosa*, the corrosion weight loss rate shows a “parabolic” growth trend with increasing flow velocity, indicating that the corrosion mechanism of X70 steel changes under the synergistic effect of microorganisms and flow velocity. Within the range of 0.3–1.0 m/s, the corrosion weight loss rate of X70 steel in seawater containing *P. aeruginosa* is higher than that in sterile seawater.

To further quantify the individual and combined effects of *P. aeruginosa* corrosion and seawater flow-induced erosion on the corrosion behavior of X70 steel, the contributions of seawater flow velocity and *P. aeruginosa* to the corrosion were calculated based on corrosion weight loss rates under different conditions, using sterile seawater immersion as the corrosion baseline.

Increment of erosion corrosion under sterile conditions:

ΔC _scouring_ = C _sterile scouring_ − C _sterile static_ (2)

Increment of corrosion under *P. aeruginosa* inoculation:

ΔC _bacteria_ = C _bacteria scouring_ − C _sterile scouring_ (3)

Increment of corrosion due to the coupling effect of *P. aeruginosa* and erosion:

ΔC _coupling_ = C _bacteria scouring_ − C _sterile static_ (4)

**Figure 12:**
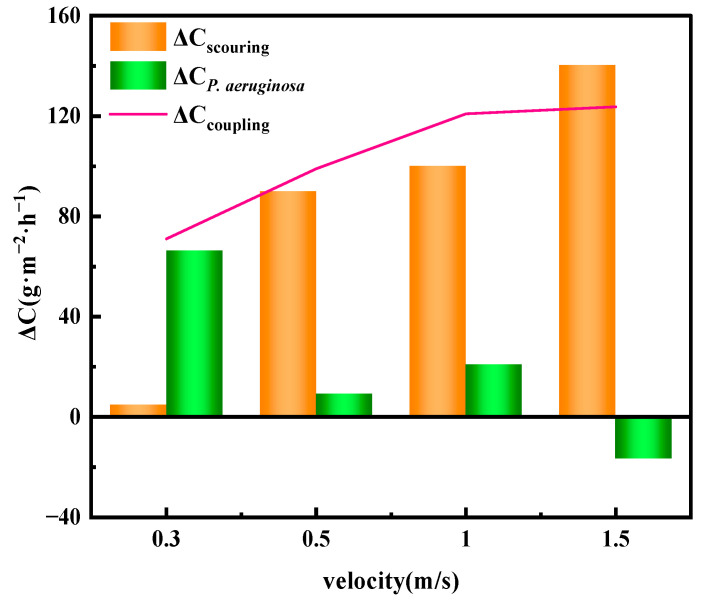
Quantification of the contribution of X70 steel to corrosion under different conditions.

ΔC _scouring_ represents the corrosion weight loss rate caused solely by scouring in sterile seawater; ΔC _bacteria_ represents the corrosion weight loss rate attributed to the presence of *P. aeruginosa*; ΔC _coupling_ denotes the synergistic effect of *P. aeruginosa* and scouring. C _sterile scouring_ is the corrosion weight loss rate measured in sterile seawater under scouring conditions; C _sterile static_ is the corrosion weight loss rate under static immersion in sterile seawater; C _bacteria scouring_ corresponds to the corrosion weight loss rate in seawater containing *P. aeruginosa* under scouring conditions. The quantified results are presented in Figure 12. All quantitative data were obtained from triplicate measurements, and the error bars are omitted in contribution plots for clarity. The results show that scouring significantly accelerates the corrosion of X70 steel, and the corrosion rate increases with increasing flow velocity. At a flow velocity of 1.5 m/s, the contribution of *P. aeruginosa* to the corrosion rate is negative, indicating that *P. aeruginosa* enhances the corrosion resistance of X70 steel under this condition. However, at flow velocities between 0.3 m/s and 1.0 m/s, *P. aeruginosa* contributes positively to corrosion. The coupling corrosion effect curve reveals that as the flow velocity increases, the synergistic corrosion weight loss due to *P. aeruginosa* and scouring becomes larger, indicating a more pronounced coupling effect.

## 4. Discussion

### 4.1. Effect of Flowing Seawater on the Corrosion of X70 Steel

The proposed reactions are schematic interpretations based on typical corrosion pathways in chloride-containing environments and were not directly confirmed by phase-sensitive techniques such as XPS or XRD in this study. The corrosion behavior of X70 steel in flowing seawater is primarily influenced by mass transfer efficiency and scouring effects. Typical corrosion morphologies in the presence of Cl^−^ ions can be clearly observed by SEM. At lower flow velocities, the flow-induced scouring accelerates the mass transfer of corrosive species, enhancing the localized attack on the steel surface [14]. Under these conditions, the overall corrosion process is governed by the efficiency of oxygen diffusion; although Cl^−^ ions tend to accumulate, the limited O_2_ supply restricts the deepening of pits. The anodic and cathodic reactions are spatially separated, manifested as follows [36,37].

Fe → Fe^2+^ + 2e^−^ (5)

O_2_ + 2H_2_O + 4e^−^ → 4OH^−^ (6)

As the flow velocity increases, the impact force on the specimen surface becomes mild and relatively uniform, allowing Cl^−^ ions to migrate toward the anodic regions. This facilitates their effective accumulation at defects, where they form chloride complexes with Fe^2+^, inhibiting repassivation and creating conditions favorable for pit propagation. FeCl_2_ reacts with OH^−^ to form Fe(OH)_2_, which, under low oxygen and locally high Cl^−^ conditions, is converted via the intermediate green rust GR(Cl^−^). Additionally, in regions with extremely high Cl^−^ concentrations, GR(Cl^−^) can be directly oxidized to form β-FeOOH.

Fe^2+^ + 2Cl^−^ → FeCl_2_ (7)

Fe(OH)_2_ + 1/2O_2_ → γ-FeOOH + H_2_O (8)

GR(Cl^−^) + O_2_ → β-FeOOH (9)

Hydrodynamic factors significantly influence the accumulation and removal of corrosion products [47].

At higher flow velocities, the impact energy of the fluid increases, generating high shear forces that overcome the resistance imposed by fluid viscosity and accelerate mass transport. When corrosion products detached from the substrate are subjected to fluid impact, they may fracture due to mechanical forces, exposing the underlying fresh metal and initiating a new corrosion cycle. The corrosion products then undergo a repeated process of formation, deposition, detachment, and reformation, leading to intensified scouring corrosion [15,16]. Under these conditions, the physical scouring effect surpasses pure electrochemical corrosion and becomes the dominant factor.

Under such high-disturbance conditions, γ-FeOOH is difficult to remain stable and undergoes an aging process of dissolution and reprecipitation, which accelerates its transformation into the thermodynamically stable α-FeOOH.

γ-FeOOH → α-FeOOH(10)

Meanwhile, under certain conditions, part of the γ-FeOOH or Fe(OH)_2_ can be transformed into black, magnetic Fe_3_O_4_.

3Fe(OH)_2_ + 1/2O_2_ → Fe_3_O_4_+ 3H_2_O (11)

Previously formed β-FeOOH is also unstable under high flow velocities, undergoing hydrolysis that releases Cl^−^ and simultaneously transforms into Fe_3_O_4_ or α-FeOOH.

β-FeOOH + H_2_O → α-FeOOH/Fe_3_O_4_ + Cl^−^ (12)

**Figure 13:**
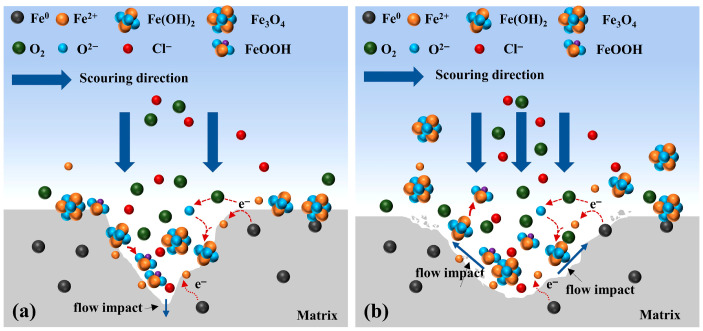
Corrosion mechanism model of X70 steel after scouring in sterile seawater (**a**): 0~1 m/s, (**b**): 1~1.5 m/s.

In summary, hydrodynamic effects play a crucial role in scouring corrosion. Under low flow velocities, the renewal rate of the corrosive medium is relatively low, resulting in slower corrosion reactions and more uniform formation of the surface film. In contrast, at high flow velocities, the hydrodynamic forces are significantly enhanced, which may lead to localized detachment of the film and exposure of fresh metal surfaces, thereby initiating new corrosion cycles. Moreover, variations in flow velocity affect the concentration distribution and mass transport of corrosive species. At high flow rates, steep concentration gradients can accelerate the corrosion process. The proposed corrosion mechanism of X70 steel under different flow conditions in the presence of Pseudomonas aeruginosa is illustrated in Figure 13.

### 4.2. Synergistic Corrosion of X70 Steel by P. aeruginosa in Flowing Seawater

**Figure 14:**
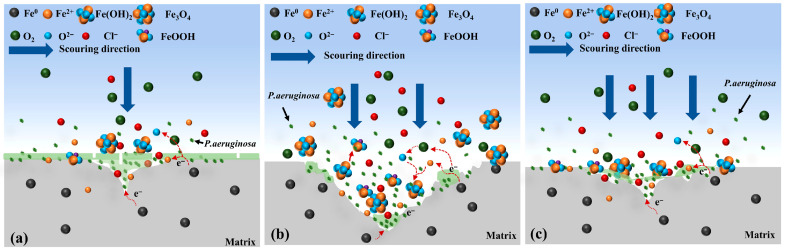
Corrosion mechanism model of X70 steel after scouring in *P. aeruginosa* seawater (**a**): 0~0.3 m/s, (**b**): 0.5~1.0 m/s, (**c**): 1.5 m/s.

Microorganisms can influence the corrosion of metallic materials either directly or indirectly, while the flow velocity governs the distribution, adhesion, and activity of microorganisms and their metabolites on material surfaces, thereby determining the efficiency of microbiologically influenced corrosion. Although calmer flow conditions may favor bacterial adhesion, extremely low flow velocity may limit the mass transfer of dissolved oxygen and nutrients, especially under low-oxygen laboratory conditions. Therefore, bacterial metabolic activity may not necessarily be maximal at the lowest flow rate. Instead, moderate flow enhances nutrient transport while maintaining biofilm stability, resulting in more pronounced MIC effects. Under completely static conditions, bacterial transport toward the metal surface is mainly diffusion-controlled, which may limit the effective redistribution and surface encounter probability of planktonic cells. A mild flow can enhance convective transport without imposing excessive shear stress, thereby increasing the probability of bacterial attachment and colonization. When the flow velocity is below 0.3 m/s, the hydrodynamic disturbance is minimal, allowing *P. aeruginosa* to colonize and proliferate on the steel surface, as shown in Figure 14a. Through respiratory activity, the bacteria can reduce or even hinder the diffusion of dissolved oxygen toward the steel surface [48,49], leading to the formation of a localized oxygen-depleted microenvironment at the metal–biofilm interface [50,51]. Although such conditions are unfavorable for the formation of corrosion products, the EPS secreted by the microorganisms locally alters the interfacial microenvironment and promotes electrochemical corrosion. Consequently, localized dissolution of the passive film occurs, and cracks initiate and propagate along grain boundaries or pre-existing defects.

Because organic carbon is available in the medium, aerobic respiration can proceed and may alter local chemistry, which is schematically expressed by Eqs:

C_6_H_12_O_6_ + 6O_2_ → 6CO_2_ + 6H_2_O (13)

CO_2_ + H_2_O ⇌ H_2_CO_3_ ⇌ H^+^ + HCO_3_^−^ (14)

Fe(III)-oxide + H^+^ + citrate^3−^ → 3Fe-citrate(aq) + H_2_O (15)

Therefore, at low flow velocities, the corrosion of X70 steel is predominantly attributed to the activity of *P. aeruginosa*.

When the flow velocity increases to 0.5–1.0 m/s, as shown in Figure 14b, the extent of MIC intensifies with increasing hydrodynamic disturbance. Higher flow velocities enhance the diffusion and transport of oxygen and nutrients in seawater, enabling their more efficient delivery into the biofilm and thereby providing favorable conditions for microbial growth and proliferation. Meanwhile, flow-induced corrosion roughens the X70 steel surface, and the corrosion products form rapidly and abundantly. When the hydrodynamic shear stress exceeds the adhesion strength between the corrosion products and the metal substrate, these products—even the passive film—can be removed, and the metabolic wastes within the biofilm can be cleared more efficiently [52]. This reduces the inhibition effect of corrosion-product accumulation on the corrosion reaction and accelerates the corrosion process. In addition, the high shear stress causes local detachment of the corrosion products or biofilm, exposing fresh metal surface and further promoting localized anodic dissolution.

Fe → Fe^2+^ + 2e^−^ (16)

O_2_ + 2H_2_O + 4e^−^ → 4OH^−^ (17)

4Fe^2+^ + O_2_ + 6H_2_O → 4FeOOH + 8H^+^ (18)

The presence of corrosion pits also provides favorable attachment sites for *P. aeruginosa*, enabling free-swimming cells to accumulate around these eroded regions. This localized aggregation further accelerates pit propagation, creating a coupled effect in which fluid-induced erosion and microbial activity synergistically promote corrosion.

When the scouring flow velocity reaches 1.5 m/s, as shown in Figure 14c, the impact force of seawater exceeds the cohesive strength of the biofilm, and the shear stress within the pits increases. This may inhibit cell adhesion and cause the biofilm to detach from the pitting areas [53]. As a result, the biofilm formed by microorganisms on the X70 steel surface becomes unstable, with physical scouring removing most of it and leaving residues only in low-shear regions such as crevices. The electron mediators are rapidly diluted by seawater, preventing indirect EET from occurring. At this stage, the biofilm formed by *P. aeruginosa* acts to protect the underlying metal [46], leading to a negative microbial contribution to the corrosion of X70 steel (as shown in Figure 12). Thus, the microorganisms exhibit a protective effect on X70 steel under these conditions.

## 5. Conclusions

(1) The corrosion behavior of X70 pipeline steel in flowing seawater is strongly influenced by flow velocity under both sterile and bacterial conditions, while the presence of *P. aeruginosa* modifies the corrosion response depending on hydrodynamic conditions.
(2) Under sterile seawater conditions, corrosion behavior is primarily influenced by mass transfer enhancement at low-to-moderate flow velocities, while flow-induced scouring becomes increasingly significant at higher velocities.
(3) In the presence of *P. aeruginosa*, corrosion behavior exhibits a flow-dependent response, with reduced corrosion rates and relatively uniform surface degradation at low flow velocities.
(4) Moderate flow velocities are associated with enhanced localized corrosion and increased pitting severity, indicating a pronounced influence of the combined effects of fluid flow and microbial presence on corrosion morphology.
(5) At higher flow velocities, strong hydrodynamic effects dominate corrosion behavior, limiting surface coverage by corrosion products and microbial films and leading to corrosion behavior primarily controlled by fluid flow.

## Figures and Tables

**Table 1:** Chemical composition of X70 pipeline steel (wt.%).

C	Si	Mn	P	S	Cr	Ti	Nb	Al	Cu	Ni	Fe
0.06	0.08	1.42	0.012	0.02	0.03	0.102	0.029	0.03	0.02	0.01	Bal

**Table 2:** Fitting results of EIS spectra under different conditions.

Corrosive Medium	Flow Velocity (m/s)	*R*_S_ (ohm·cm^2^)	*Q*,*Y*_0_ (S·s^n^/cm^2^)	*n*_1_	*R*_f_ (ohm·cm^2^)	*Q*_dl_, *Y*_0_ (S·s^n^·/cm^2^)	*n*_2_	*R*_ct_ (ohm·cm^2^)
X70 steel in sterile seawater	0	8.80	3.82 × 10^−4^	0.89	─	─	─	7760
	0.3	7.48	1.79 × 10^−4^	0.91	1482	0.20 × 10^−3^	0.93	5361
	0.5	7.25	7.61 × 10^−4^	0.80	441	1.57 × 10^−3^	0.77	619
	1.0	15.74	31.80 × 10^−4^	0.88	814	2.70 × 10^−3^	0.95	227
	1.5	15.33	54.74 × 10^−4^	0.84	46	3.45 × 10^−3^	0.81	52
X70 steel in seawater containing *P. aeruginosa*	0	7.12	3.13 × 10^−4^	0.91	─	─	─	8020
	0.3	7.76	4.48 × 10^−4^	0.82	537	0.84 × 10^−3^	0.84	1116
	0.5	7.35	7.68 × 10^−4^	0.92	1083	1.54 × 10^−3^	0.90	228
	1.0	9.07	26.41 × 10^−4^	0.80	222	4.17 × 10^−3^	0.79	150
	1.5	9.72	39.86 × 10^−4^	0.75	149	2.63 × 10^−3^	0.73	202

**Table 3:** Electrochemical parameters fitted from the potentiodynamic polarization curves under different conditions.

Corrosive Medium	Flow Velocity (m/s)	*I*_corr_ (µA/cm^2^)	*E*_corr_ (mV_SCE_)	*Β*_a_ (mV/dec)	*Β*_c_ (mV/dec)
X70 steel in sterile seawater	0	7.49	−783.24	375.76	−226.86
	0.3	9.22	−698.61	89.84	−142.62
	0.5	14.23	−638.76	46.01	−203.77
	1.0	81.97	−599.37	181.36	−426.11
	1.5	128.96	−828.44	362.93	−173.39
X70 steel in seawater containing *P. aeruginosa*	0	4.79	−788.65	220.32	−170.25
	0.3	12.24	−796.18	316.07	−130.13
	0.5	25.83	−749.58	228.61	−184.81
	1.0	96.42	−727.37	118.23	−202.45
	1.5	87.72	−745.87	206.69	−236.04

## Data Availability

The original contributions presented in this study are included in the article. Further inquiries can be directed to the corresponding author.
